# Hepatitis B Seroprevalence and Risk Factors in Adult Population of Chaharmahal and Bakhtiari Province in 2013

**DOI:** 10.5812/hepatmon.17389

**Published:** 2014-05-03

**Authors:** Masoumeh Moezzi, Reza Imani, Nasser Khosravi, Behrouz Pourheidar, Forouzan Ganji, Ali Karimi

**Affiliations:** 1 Department of Community Medicine, Shahrekord University of Medical Sciences, Shahrekord, IR Iran; 2 Department of Infectious Diseases, Shahrekord University of Medical Sciences, Shahrekord, IR Iran; 3 Central Disease Control Unit, Shahrekord University of Medical Sciences, Shahrekord, IR Iran; 4 Department of Microbiology, Shahrekord University of Medical Sciences, Shahrekord, IR Iran

**Keywords:** Hepatitis B, Hepatitis B Surface Antigens, Seroepidemiologic Studies, Risk Factors

## Abstract

**Background:**

Hepatitis B virus is one of the important viral causes of liver inflammation with high worldwide prevalence and important hepatic and extra hepatic complications.

**Objectives:**

The aim of this study was to investigate the prevalence and risk factors of hepatitis B in Chaharmahal and Bakhtiari province, Iran.

**Patients and Methods:**

For this descriptive, analytical, population-based study, 3000 participants older than 15 years were enrolled according to the clustering method. After obtaining written informed consent and taking required blood samples, we gathered data on demographic status and probable transmission routes of disease using questionnaire between 2012 and 2013. The data was analyzed using SPSS software (descriptive parameters and chi-square). P value below 0.05 was considered as statistically significant.

**Results:**

The mean age of participants was 38.4 ± 16.3. The seroprevalence rate of hepatitis B was found to be 1.3% (95% CI, 0.95%-1.81%). Prevalence of HBeAg among HBsAg positive participants was 2.5% (only 1 of 40). Seroprevalence was higher in male group (2.5 times higher than women), age group of over 55 years, farmers, and non-public occupations. Positive seroprevalence was associated with a history of renal disease, familial transmission, transfusion, surgery in hospital, circumcision, contact with hepatitis B infected individuals, imprisonment, intravenous (IV) drug abuse, and smoking (P < 0.05). Nevertheless, the highest odds ratio (OR) was obtained for history of renal disease (OR = 7.64: 3.01-18.4), followed by imprisonment (OR = 5.4: 1.86 -15.7) and IV drug abuse (OR = 5.68: 1.3-24.7).

**Conclusions:**

Chaharmahal and Bakhtiari province could be categorized as a low endemic region for hepatitis B infection, with a seroprevalence similar to that in other provinces of western Iran. Vaccination seems to influence its decrease, especially in adolescents and youth. More surveillance and attention to risk factors are suggested to identify high-risk groups and to implement vaccination.

## 1. Background

Hepatitis is a prevalent liver inflammation, which develops because of different reasons including various viruses, medications, alcohol, lipid tissue replacement, and some others. Hepatitis B virus is one of the important viral causes, which its significance is due to worldwide, high prevalence and important hepatic and extra hepatic complications (1). Currently, three-fourths of the world populations live in regions with high prevalence of hepatitis B and approximately one million people each year die from its complications, such as cirrhosis and hepatocellular carcinoma. Global prevalence of hepatitis B is 5%; hence, it is considered as one of the most prevalent infectious diseases (2-4).

Prevalence of hepatitis B infection in general population of Iran was reported 1.7% (0-3.9%) in 1996 (5). According to the previous research, Iran was categorized as a country with a moderate (2-8%) prevalence (5-8), but latest studies showed prevalence transition from moderate to low (0.1-2%) due to vaccination in the recent years (9-17). 

In world regions with low hepatitis B prevalence, the highest prevalence is observed in adolescents and young adults, and the main transmission routes are sexual contact, transfusion of infected blood, and unsafe injections. Vaccination has decreased the childhood prevalence and could decrease transmission and prevalence in adults who are more likely to develop the infection, as well (18-22). 

However, hepatitis B infection is an important health problem throughout the world and Iran, and is still hyperendemic in some regions of the world; its control comprises a large portion of countries health expenditure per capita (1).

## 2. Objectives

As mentioned above, general prevalence of this disease has been already determined in some provinces of Iran, but it has not been determined in Chaharmahal and Bakhtiari province, which requires investigation to help policymaking. Therefore, we conducted this population-based study to investigate the seroprevalence of hepatitis B based on HBsAg positivity and risk factors for hepatitis B in this province.

## 3. Patients and Methods

Adults older than 15 years living in urban and rural areas of Chaharmahal and Bakhtiari were the sample population of this descriptive, analytical, population-based study. According to statistical advice and with assumption of 2% seroprevalence of hepatitis B, confidence interval of 95%, and error rate of 25% regarding participants’ weight in similar studies, and considering 800000-individual population of the province (672535 individuals over 15 years old), 3000 individuals were enrolled. The ratio of province total population to participants (weight) was 266, which is considered appropriate compared to similar studies. The method of sampling was cluster sampling, including 50 clusters (of 60 individuals each) (32 urban, 18 rural) from seven counties of the province (Table 1). The clusters and proportion of urban and rural population of each cluster were considered according to estimates of the latest national statistical census at the time of conducting this investigation. The inclusion criteria were being 15 years and/or older and consent to participate. Anyone who did not agree with blood sampling or any household that was absent in two times of referring were eliminated from the study and then another individual or family was replaced according to the study protocol. The number of individuals who did not agree with blood sampling was negligible due to counseling program and obtaining their consent. Therefore, it could not lead to bias in our study. This study holds ethics code of 90-2-6 obtained from the Ethics Committee of University. To implement the program, three teams of interview, blood sample taking, and laboratory were formed. Sample taking was performed by interview team referring to houses, showing identification card, explaining the pur--pose of the project, and asking the presence of qualified individual(s) in their house. Then, the questionnaire was filled after obtaining consent. The qualified individuals in urban areas were referred to central laboratory of each county for blood sampling and in rural areas, this was exclusively performed in Health Houses. In total, 5mL blood samples were taken from qualified individuals and centrifuged in the same place, therefore the obtained 2-3 mL serum, as coded, was referred to the laboratory team. The duration of gathering data was between 2012 and 2013. In this study, we intended to assess HBV infection seroprevalence, confirmed by detection of blood specimen with positive results for HBsAg. In addition, HBeAg was tested to assess the infectivity rate. In fact, we did not intend to distinguish between acute and chronic infection, so anti HBcAb was not checked and it would be performed in future studies. For HBsAg and HBeAg test, Delaware Kit (Common Market) was used. Data was entered SPSS 19 software, and statistical analysis was performed using descriptive statistics and chi-square test. The level of significance was considered 0.05.

**Table 1.** The Clusters Under Study Based on Urban and Rural Population in Chaharmahal and Bakhtiari Counties ^a^

	The Province population (total/Over 15 years)	Population (Shahrekord/Kiar)	Boroujen	Ardal	Farsan	Lordegan	Kouhrang
**Population**	(857910/672535)	(325188/9192)	115286	54464	90980	177277	35520
**Clusters**							
**U**	32	25	6	-	-	1	-
**R**	18	7	1	1	3	4	2

^a^ Abbreviations: SH, Shahrekord; K, Kiar; U, urban; R, rural.

## 4. Results

The mean age of participants was 38.4 ± 16.3 (15-90). In total, 37% were male and 25% single. To examine the seroprevalence of hepatitis B, HBsAg test was performed. Of 3000 participants, 40 were seropositive and the prevalence of HBsAg was found to be 1.3% (95% CI, 0.95%-1.81%). HBeAg-positivity was also examined and only one case (of total 40 cases [2.5%]) had positive findings. 

Table 2 shows some demographic criteria. The highest prevalence was obtained in Boroujen (2%), followed by Shahrekord and Lordegan (both 1.3%). The prevalence in male participants was 2.5 times higher than female ones. For age, the highest prevalence was obtained in age group of 55-64 years old and over 64 years old. The seroprevalence in married participants was obtained 1.5% and in single 0.5%. For ethnicity, the prevalence in Turks was 1.9% and in Fars 1.5%, which were higher compared to Lurs (0.5%). For occupation, the highest prevalence was obtained in participants of non-public occupations and farmers. The prevalence in urban areas was 1.5% and in rural areas 0.6%. For educational level, the highest rate was obtained in illiterate participants (1.9%), and those able to only read and write (1.6%), decreasing as educational level was higher; hence, the prevalence in diploma and higher than diploma holders was 0.9% and in BSc/MSc and higher than BSc/MSc holders 0.7%.

There was significant association between the prevalence and disease history, first-degree relative infected with hepatitis, blood transfusion, history of operation in hospital, circumcision, and history of contact with hepatitis B infected individuals, imprisonment history, intravenous (IV) drugs abuse history, and smoking history. Seroprevalence in participants with tattoo history was 2%, higher compared to participants with no tattoo history (1.2%), but the difference was not significant (P = 0.07). The seroprevalence in participants with history of visiting the Persian Gulf countries was two times higher than others without this history, but the difference was not significant (P = 0.06). The highest odds ratio was obtained for renal disease history, followed by IV drugs abuse history and imprisonment history (Table 3). The association between the prevalence and jaundice history, phlebotomy, dental procedures, endoscopy, dialysis, acupuncture, organ transplantation, scarification, shared syringe, suspicious sexual contact, and breast feeding was not significant.

**Table 2.** Prevalence of Hepatitis B for Demographic Characteristics ^a^

	Total Number	Positive Cases No.	Prevalence, %	P value ^a^ Chi-Square
**County**				0.09 Fisher’s Exact Test
Shahrekord	1680	23	1.3	
Boroujen	420	11	2	
Farsan	120	0	0	
Lordegan	300	4	1.3	
Ardal	60	0	0	
Kouhrang	180	0	0	
Kiar	240	2	0.8	
**Gender**				0.007
Male	1111	23	2	
Female	1889	17	0.8	
**Age, y**				0.01
15-24	708	4	0.5	
25-34	766	8	1	
35-44	518	4	0.7	
45-54	456	9	1	
55-64	384	11	2	
> 64	161	4	2	
**Occupation**				0.01 Fisher’s Exact Test
Civil Servant	151	1	0.6	
Non-Public	506	18	3	
Student	340	1	0.2	
Soldier	12	0	0	
Housewife	1495	13	0.8	
Retired	102	2	1.9	
Jobless	278	3	1	
Farmer	81	2	2	
Poulterer	11	0	0	

^a^ At 0.05 level, P is significant.

**Table 3.** Prevalence of Hepatitis B for Risk Factors ^a^

Risk factor	Total Number	Positive Cases Number	Prevalence, %	OR	CI	P Value ^b^ Chi-Square
**Disease ^c^**						
Diabetes mellitus	97	3	3	3.32	0.9-11.35	0.002 Fisher’s Exact Test
Renal	103	7	6	7.64	3.01-18.4	
Hepatic	28	0	-	-	-	
Gastrointestinal	171	4	2	2.49	0.84-7.35	
Hematology	29	0	-	-	-	
Others	350	5	1.4	1.5	0.56-4	
None ^d^	2210	21	0.9	-	-	
**Relative infected with virus**						0.008
Yes	93	5	5.3	4.6	1.7-12.03	
No	2871	35	1.2			
**Transfusion**						0.008
Yes	72	3	4	3.35	1.01-11.1	
No	2894	37	1			
**Operation in hospital**						0.04
Yes	1236	23	1.8	1.9	1.1-3.5	
No	1728	17	0.9			
**Tattoo**						0.07
Yes	307	8	2	2.19	1-4.79	
No	2651	32	1.2			
**Circumcision**						0.01
Yes	1063	23	2	2.31	1.14-4.6	
No	1270	12	0.9			
**Contact with the infected**						0.004
Yes	120	6	5	4.45	1.82-10.83	
No	2824	33	1			
**Imprisonment**						0.001
Yes	63	4	6	5.41	1.86-15.7	
No	2912	36	1			
**IV drug abuse**						0.009
Yes	29	2	6	5.68	1.3-24.7	
No	2952	38	1			
**Visiting Persian Gulf countries**						0.06
Yes	313	8	2	2.15	0.98-4.7	
No	2657	32	1			
**Smoking**						0.001
Yes	417	14	3	3.36	1.74-6.5	
No	2545	26	1			

^a^ Abbreviations: OR, odds ratio; CI, confidence interval.

^b^ At 0.05 level, P is significant.

^c^ Reference is HBsAg-negative.

^d^ This parameter is set to zero.

## 5. Discussion

The seroprevalence of hepatitis B in Chaharmahal and Bakhtiari province was estimated 1.3%, indicating that this province could be categorized as a low prevalence area. Only one case (of total 40 [2.5%]) was HBeAg-positive, which could represent the disease low capability of transmission.

Hepatitis B prevalence in Iran has been reported for different regions. In a national study of Iran on a 39841-individual population 2 to 69 years old in 1996, the mean hepatitis B prevalence was estimated 1.7% (0-3.9%) (5). In the study of Nahavand, western Iran, in a 1824-individual population over 5 years old, HBsAg prevalence was 2.3%, of which 9.5% were HBeAg-positive (9). In a systematic review by Alavian et al. investigating 14 studies conducted between 2001 and 2007 in seven provinces of Iran, general hepatitis prevalence was found to be 2.14% (men, 2.55%; women, 2.03%). The prevalence in Golestan was 6.3%, Tehran 2.2%, Eastern Azarbaijan 1.3%, Hamadan 2.3%, Isfahan 1.3%, Kermanshah 1.3%, and Hormozgan 2.4% (10). Of studies published after 2007, those conducted in Kohgiloyeh and Boyerahmad with 1.2% prevalence (11), in Kurdistan in 2012 on 1613 six- to 65-years-old individuals with 0.8% prevalence (12), in Zahedan investigating 2587 individuals, with 2.5% prevalence (13), in Sistan and Baluchistan with 3.38% prevalence (14), and in Qom with 1.3% prevalence (15) could be mentioned. Examining geographical distribution according to these results indicates that the highest prevalence was observed in northeastern, and the prevalence is lower in central and western Iran The result of the present study is similar to those relevant to the provinces in western Iran.

In other countries, various prevalence rates were found. In a study in Pakistan on 7000 individuals (200-250 households) in 2008, hepatitis B prevalence was found 2.5% and HBeAg-positivity prevalence in seropositive cases was reported 14.5% (23). In China, a 2.4% prevalence was reported (24), in Singapore in 2005 a 2.88% prevalence (25), and in Bangladesh a 6.5% prevalence (26). It seems that the prevalence in Iran is less compared to other developing countries. Despite the difference among different regions, Iran’s prevalence has been undergoing a declining trend. In this province, the prevalence has followed the pattern in western provinces. Despite, definite judgment regarding the prevalence trend is impossible due to lack of data on population-based prevalence prior to vaccination. It seems that the national vaccination plan has decreased the prevalence.

Regarding the risk factors and the factors affecting the disease transmission, sexual contacts, transfusion of infected blood and/or blood products, IV drugs abuse, shared syringe use, and unsafe injections are usually considered as the most important routes (18-22). Despite the fact, the results of different studies are various. In this study, of demographic factors gender, age, and occupation were found to have significant association with prevalence. Although the prevalence was higher in men, age group of over 55 years old, participants with non-public occupations and farmers, married ones, those living in urban areas, and with low educational level. Factors of renal disease history, first-degree relative infection with hepatitis B, blood transfusion, history of operation in hospital, circumcision, history of contact with a hepatitis B-infected patient, imprisonment history, IV drug abuse history, and smoking had a significant association with disease seroprevalence.

In a national study in 1996, in contrast to the present study, the prevalence in rural areas was higher compared to urban areas, but similar to this study, the prevalence in men, age group of 50 to 59 years old, farmers, married ones, and low educational level was higher, and no association with clinical symptoms was observed (5). In the study of Nahavand, the prevalence rate was significantly associated with surgery history, imprisonment history, and age (9). The results of study in Kohgiloyeh and Boyerahmad suggested the effect of residency, educational level, and IV drug use (11). In a study performed in Kurdistan, being 46 to 65 years old and married were reported as effective factors, but there was no difference between the two genders. In addition, blood transfusion, addiction, hospitalization history and imprisonment had no significant association with disease prevalence (12). In a study in Zahedan, there was an association between seroprevalence and age, gender, and marital status and, consistent with the present study, the prevalence was higher in age group of over 55 years old, men, and the married ones. Nevertheless, no association was observed between disease and surgery history, educational status, smoking, and occupation (13). In a study performed in Qom, consistent with the present study, the prevalence was higher in men and was significantly associated with age and educational level (15). 

Some studies conducted on blood donor groups and/or the groups at risk like prisoners and IV drug abusers indicated more likelihood of the disease in individuals with low educational level and sexual contact history, married ones, and IV drug abusers in Iran and horizontal transmission has been noted more prevalently compared to vertical, as well (20-22).

In a study in Pakistan the prevalence in men, married ones, and IV drug abusers, low educational level, and public and non-public, outside home occupation holders was higher (23). In a study in China, the prevalence increased as age increased and the prevalence in men was higher compared to women (24). In a study in Bangladesh, an association was observed with being married, and surgery history, getting ear and nose pierced, and circumcision were reported as significant risk factors (26).

Generally, risk factors of IV drug abuse history, imprisonment history, and familial or non-familial relationship with infected ones have been identified as main risk factors for the disease transmission, which predictably had the highest odds ratio in the present study, as well. 

Since chronic renal failure is considered as a high risk group for HBV infection (27), significant odds ratio in individuals with renal disease history compared to those with other diseases was not unexpected; perhaps, it is due to immunodeficiency associated with this disease. However, study of Guilan, Iran indicated a relatively low prevalence of HBV infection in patients with end stage renal disease. Moreover, vaccination prior to chronic hemodialysis setting and antiviral treatment were offered as the reasons for this prevalence rate (28), but the investigated individuals in the present study were enrolled from general population, who are different from the samples in Guilan study (28).

Seroprevalence of hepatitis B in Chaharmahal and Bakhtiari was found to be 1.3%; hence, it could be considered as a region with low prevalence similar to other western provinces. Vaccination could be considered as a factor effective to decrease its prevalence; particularly among adolescents and youth (although less proportion of male to female participants [40% vs. 60%] should be considered). Appropriate care and surveillance to find the groups at risk regarding the identified risk factors and vaccination implementation for them could be helpful in further decrease in hepatitis B prevalence.
